# Association Between Parental Educational Attainment and Youth Outcomes and Role of Race/Ethnicity

**DOI:** 10.1001/jamanetworkopen.2019.16018

**Published:** 2019-11-22

**Authors:** Shervin Assari, Cleopatra H. Caldwell, Mohsen Bazargan

**Affiliations:** 1 College of Medicine, Department of Family Medicine, Charles R. Drew University of Medicine and Science, Los Angeles, California; 2 Department of Health Behavior and Health Education, University of Michigan School of Public Health, Ann Arbor; 3 Center for Research on Ethnicity, Culture, and Health, University of Michigan School of Public Health, Ann Arbor; 4 Department of Family Medicine, University of California, Los Angeles, Los Angeles

## Abstract

**Importance:**

The concept of minorities’ diminished returns refers to the smaller protective effects of educational attainment for racial and ethnic minority groups compared with those for majority groups.

**Objective:**

To explore racial and ethnic differences in the associations between parental educational attainment and youth outcomes among US adolescents.

**Design, Setting, and Participants:**

A cross-sectional study was performed of 10 619 youth aged 12 to 17 years who were participants at wave 1 of the Population Assessment of Tobacco and Health (PATH) study, a nationally representative survey, in 2013. Data analysis was performed from August to October 2019.

**Main Outcomes and Measures:**

The dependent variables were youth tobacco dependence, aggression, school performance, psychological distress, and chronic medical conditions. The independent variable was parental educational attainment. Age and sex of the adolescents and marital status of the parents were the covariates. Race and ethnicity were the moderating variables. Logistic regression was used for data analysis.

**Results:**

Among the participants, 5412 (51.0%) were aged 12 to 15 years, and 5207 (49.0%) were aged 16 to 17 years; 5480 (51.7%) were male. For non-Hispanic white youth, as parental educational attainment increased, there were stepwise reductions in the prevalence of tobacco dependence (13.2% vs 6.9% vs 2.7%), aggression (37.9% vs 34.8% vs 26.1%), low grade point average (84.2% vs 75.6% vs 53.3%), and chronic medical conditions (51.7% vs 50.8% vs 43.9%), but there was not such a trend for psychological distress (43.7% vs 48.6% vs 41.0%). Interactions were significant between Hispanic ethnicity and parental education on tobacco dependence (OR, 3.37 [95% CI, 2.00-5.69] for high school graduation; OR, 5.40 [95% CI, 2.52-11.56] for college graduation; *P* < .001 for both), aggression (OR, 1.41 [95% CI, 1.09-1.81]; *P* = .008 for high school graduation; OR, 1.59 [95% CI, 1.14-2.21]; *P* = .006 for college graduation), and psychological distress (OR, 1.50 [95% CI, 1.05-2.13]; *P* = .03). Black race showed an interaction with college graduation on poor school performance (OR, 2.00 [95% CI, 1.26-3.17]; *P* = .003) and chronic medical conditions (OR, 1.56 [95% CI, 1.14-2.14]; *P* = .005). All these findings suggest that the protective associations between high parental educational attainment and youth development might be systemically smaller for Hispanic and black youth than for non-Hispanic youth.

**Conclusions and Relevance:**

Although high parental educational attainment is associated with better outcomes for youth, this association is systemically less significant for Hispanic and black than non-Hispanic white youth. The result is an increased health risk in youth from middle class black and Hispanic families. Given the systemic pattern for outcomes across domains, the diminishing returns of parental educational attainment may be due to upstream social processes that hinder ethnic minority families from translating their capital and human resources into health outcomes.

## Key Points

**Question:**

Is parental educational attainment associated with the same amount of health benefits for various ethnic groups?

**Findings:**

In this cross-sectional study of 10 619 youth aged 12 to 17 years, parental educational attainment was associated with fewer health benefits for Hispanic youth compared with their non-Hispanic counterparts.

**Meaning:**

These findings suggest that elimination of ethnic health disparities requires more than equalizing socioeconomic resources across ethnic groups.

## Teaser

This cross-sectional study uses data from the Population Assessment of Tobacco and Health survey to explore racial and ethnic differences in the associations between parental educational attainment and youth outcomes among US adolescents.

## Introduction

Race- and ethnicity-associated health disparities among youth are a public health concern in the United States.^1,2,3^ The worse health outcomes of black and Hispanic youth compared with non-Hispanic youth are attributed, at least in part, to lower socioeconomic status (SES), particularly parental educational attainment.^4,5^ Outcomes tend to be worse for black and Hispanic youth than for non-Hispanic white youth, possibly because of high levels of socioeconomic adversity.^6^ These disparities can be seen across domains, such as health risk behaviors,^7,8^ aggression,^7,8^ school performance,^5,7^ psychological well-being,^9,10^ and physical health.^2^ For example, chronic medical conditions (CMCs), such as asthma^11^ and diabetes,^12,13^ are more common among black and Hispanic than non-Hispanic white youth. Disparities in school achievement are also well described in the United States.^14^

Because race and ethnicity closely overlap with SES indicators (eg, educational attainment),^2,15^ at least some of the racial and ethnic disparities in youth outcomes traditionally have been attributed to SES inequalities across racial and ethnic groups.^16,17,18^ Recent research,^19,20^ however, has introduced minorities’ diminished returns (MDRs) as an overlooked mechanism behind racial and ethnic health disparities. The concept of MDR refers to weaker-than-expected associations between SES indicators, particularly educational attainment, and tangible health outcomes for racial and ethnic minority groups compared with the majority group.^19,20^ Minorities’ diminished returns emphasize that some of the racial and ethnic disparities are beyond group differences in access to SES resources but instead are due to the group differences in their outcomes. As such, MDRs provide a paradigm shift for understanding the underlying mechanisms behind the racial and ethnic disparities within the middle class.^19,20^ Minorities’ diminished returns are also of interest because they provide an explanation for why racial and ethnic disparities have persisted despite enormous investments seeking to eliminate them.^21,22,23^

A growing body of research^24,25,26,27,28,29^ has suggested that high parental educational attainment is associated with smaller health gains among non-Hispanic black youth than among non-Hispanic white youth. Diminished returns of parental SES on self-rated health,^25^ depression,^30,31^ anxiety,^32^ impulse control,^27^ school performance,^33^ school attainment,^24^ school bonding,^34^ obesity,^29,35^ asthma,^36^ attention-deficit/hyperactivity disorder,^37^ health care use,^26^ and smoking^38^ are shown for non-Hispanic black compared with non-Hispanic white families. All these results, however, are derived from studies comparing non-Hispanic black and non-Hispanic white youth, and very limited knowledge exists on MDRs for Hispanic youth. Thus, there is a need to extend the MDR literature from black to Hispanic families.

Building on prior research on MDRs^19,20^ and using a nationally representative sample of youth in the United States,^39,40^ this study compares racial and ethnic groups of youth for the association between parental educational attainment and the following 5 youth outcomes: tobacco dependence, aggression, low grade point average (GPA), psychological distress, and CMCs. The present study expands the MDRs literature from studies of black adults,^41,42,43^ black youth,^27,28,29^ and Hispanic adults^44,45,46^ to Hispanic youth, and it tests whether MDRs are robust regardless of ethnicity and outcome. If MDRs are due to upstream social phenomena (eg, marginalization and differential treatment), we should expect a general pattern of MDRs across outcomes among both black and Hispanic individuals.

## Methods

### Design and Settings

This cross-sectional study is a secondary analysis of data from wave 1 of the Population Assessment of Tobacco and Health (PATH) study.^39,40^ Funded by the National Institutes of Health and the Food and Drug Administration, PATH is a state-of-the-art study on tobacco use of US youth. PATH has enrolled 13 650 people aged 12 to 17 years who are representative of US youth. Wave 1 of PATH youth data were collected in 2013 to 2014. The analytical sample for this study was limited to 10 619 youth who were either Hispanic or non-Hispanic black or white. Any individual of another racial or ethnic group, such as Asian American, Native American/Alaska Native, mixed race, or unknown race/ethnicity, was excluded.

All youth participants in the PATH study provided written assent. Their parents or guardians also provided written informed consent. The institutional review board of Westat approved the PATH study protocol. Charles Drew University of Medicine and Science institutional review board exempted the current secondary analysis from a full review. This study follows the Strengthening the Reporting of Observational Studies in Epidemiology (STROBE) reporting guideline.^47^

### Sample and Sampling

The PATH study’s youth samples in wave 1 were the civilian, noninstitutionalized, US population aged 12 to 17 years. The PATH study used a 4-stage stratified probability sampling to recruit participants. At first, 156 primary sampling units were selected. Primary sampling units were counties or a group of counties. The second stage formed and sampled smaller geographical segments in each primary sampling unit. The third stage involved sampling of residential addresses, using the US Postal Service Computerized Delivery Sequence Files. The fourth stage was the selection of one participating youth from the household with an eligible youth. The response rate of the participants at wave 1 of the PATH study was 78.4%.

### Study Variables

The study variable roles are as follows: race and ethnicity as the moderators, parental educational attainment as the independent variable, youth outcomes as the dependent variables, and demographic factors (age, sex, and family structure) as the covariates. All the study variables were measured at the individual level. Publicly available data do not provide information on zip code; thus, we could not geocode our data in order to use higher-level SES indicators.

#### Race

Race (1, black vs 0, white) was self-identified. Race was a dichotomous variable.

#### Ethnicity

Ethnicity (1, Hispanic vs 0, non-Hispanic) was self-identified. Ethnicity was a dichotomous variable.

#### Parental Educational Attainment

Parents reported their educational attainment. Parental educational attainment was a 3-level nominal variable: less than high school graduate, high school graduate or more but not college graduate, and college graduate.

#### Demographic Factors

Age, sex, and family structure were demographic covariates. Age was a dichotomous variable (0, 12 to 15 years old vs 1, 16 to 17 years old). Family structure was a dichotomous variable (1, married vs 0, unmarried) and calculated according to parents’ marital status.

#### Tobacco Dependence

Tobacco dependence was calculated using the following 3 items: time to wanting to use first tobacco product after waking, ever having strong cravings to use tobacco, and ever feeling that one really needed to use tobacco. Responses to these items ranged from 1 (low) to 5 (high). We operationalized tobacco dependence as a dichotomous outcome (1, any level of dependence vs 0, no dependence). This measure is a standard measure and is used to model tobacco dependence in youth.^48^

#### Aggressive Behavior

Two items were used to measure aggressive behaviors. Participants were asked “When was the last time you did the following 2 or more times: were a bully or threatened other people and started physical fights with other people?” Items were positively correlated (*r* = 0.30). Item responses ranged from 1 to 4: 1, never; 2, over a year ago; 3, 2 to 12 months ago; and 4, past month. We calculated a sum score that ranged from 2 to 8, with a higher score indicating more aggressive behaviors. These items are taken from the externalizing behaviors component of the Global Appraisal of Individual Needs–Short Screener.^49^ This measure predicts future risk of arrest or incarceration within the last 12 months. This variable has been previously used as a categorical variable reflecting high-risk individuals, with very good predictive validity.^50^ Studies have shown that the Global Appraisal of Individual Needs–Short Screener instrument has adequate internal consistency, as well as overall and subscale construct validity.^49^

#### Low Grade Point Average

Participants were asked “What is your current overall GPA?” The answers were “mostly *A*’s,” “*A*’s and *B*’s,” “mostly *B*’s,” “*B*’s and *C*’s,” “mostly *C*’s,” “*C*’s and *D*’s,” “mostly *D*’s,” “*D*’s and *F*’s,” and “mostly *F*’s.” Low GPA was treated as a binary outcome (0, mostly As vs 1, any other status). Self-reported GPA and grades are well-established outcomes and are commonly used to measure school performance. Validation studies have shown that, overall, the self-reported GPA has very low absolute over- and underreporting, which emphasizes its validity across grade levels and subject areas.^51^

#### Psychological Distress

Psychological distress was measured using the following 4 items. “When was the last time you had significant problems with the following: feeling very trapped, lonely, sad, blue, depressed, or hopeless about the future; sleep trouble, such as bad dreams, sleeping restlessly, or falling asleep during the day; feeling very anxious, nervous, tense, scared, panicked, or like something bad was going to happen; and becoming very distressed and upset when something reminded you of the past?” Item responses ranged from 1 to 4 as follows: 1, never; 2, over a year ago; 3, two to twelve months ago; and 4, past month.^52^ We calculated a sum score that ranged from 4 to 16, with a higher score indicating more psychological distress (Cronbach α = 0.82). These items are taken from the internalizing behavioral component of the Global Appraisal of Individual Needs–Short Screener instrument,^49^ which was previously used to categorize youth as low vs high risk. This simplified classification has good predictive strength,^50^ as well as internal consistency and overall and subscale construct validity.^49^

#### Chronic Medical Conditions

To measure CMCs, parents were asked whether any physician has ever told them that their child has any of the following 7 CMCs: hypertension, high cholesterol, asthma, bronchitis or pneumonia, attention-deficit/hyperactivity disorder, dental problem, and diabetes. Parent reports provide valid information regarding CMCs, although some bias due to self-report measurement is expected. We treated CMCs as a binary outcome, where 1 equals 1 or more CMCs regardless of their type, and 0 equals no CMCs.

### Statistical Analysis

We analyzed the data using SPSS statistical software version 23.0 (IBM). To analyze the PATH data, survey weights that are due to the sampling design (clustered stratified sampling) were applied. Because survey weights were considered, the results are representative of the US youth population. Taylor series linearization was used to re-estimate the variance of the variables. For our data analysis, we first examined the distribution of our variables. We did not perform linear regression because 3 of our variables were not linearly distributed (ie, tobacco dependence, aggressive behaviors, and number of CMCs). We also tested the assumption of homoscedasticity (eg, random distribution of error terms), which failed for tobacco dependence, aggressive behaviors, and number of CMCs. Because we had 5 outcomes with different distributions, because it was essential for us to use the very same multivariable modeling approach to all outcomes (for comparability of MDRs across outcomes), and because we could not perform linear regression for 3 of our outcomes, we chose to operationalize all our outcomes as dichotomous. Thus, for multivariable analysis, we applied binary logistic regressions to all our outcomes (ie, tobacco dependence, aggression, low GPA, psychological distress, and CMCs). We ran 2 logistic regression models per outcome, all in the pooled sample that included white, black, Hispanic, and non-Hispanic participants. The first model did not have any interaction terms. The second model included 4 interaction terms between race and ethnicity with parental educational attainment (high school and college graduation). From our logistic regression models, we reported odds ratios (ORs), 95% CIs, and *P* values (2-sided χ^2^ tests). *P* < .05 was considered statistically significant. Data analysis was performed from August to October 2019.

## Results

### Descriptive Statistics of the Participants

Table 1 summarizes descriptive statistics for the overall sample. This study included 10 619 US youth who were aged 12 to 17 years. The sample was almost half male (5480 [51.7%]) and half female (5113 [48.3%]), and also half 12 to 15 years old (5412 [51.0%]) and half 16 to 17 years old (5207 [49.0%]). In total, 1982 youth (18.7%) were black and 8637 (81.3%) were white. Among all participants, 8236 were non-Hispanic (77.6%) and 2383 were Hispanic (22.4%).

**Table 1.** Descriptive Statistics in the Overall Sample and by Race and Ethnicity

Variable	All Participants, No. (%) (N = 10 619)	Ethnicity, No. (%)		*P* Value^a^	Race, No. (%)		*P* Value^a^
		Non-Hispanic (n = 8236)	Hispanic (n = 2383)		White (n = 8637)	Black (n = 1982)	
Sex							
Female	5113 (48.3)	3967 (48.3)	1146 (48.3)	≥.05	4166 (48.3)	947 (48.0)	≥.05
Male	5480 (51.7)	4252 (51.7)	1228 (51.7)		4456 (51.7)	1024 (52.0)	
Age, y							
12-15	5412 (51.0)	4108 (49.9)	1304 (54.7)	≥.05	4399 (50.9)	1013 (51.1)	≥.05
16-17	5207 (49.0)	4128 (50.1)	1079 (45.3)		4238 (49.1)	969 (48.9)	
Family structure							
Nonmarried parents	3820 (36.0)	2867 (34.8)	953 (40.0)	<.05	2618 (30.3)	1202 (60.6)	<.05
Married parents	6792 (64.0)	5365 (65.2)	1427 (60.0)		6012 (69.7)	780 (39.4)	
Parental educational attainment							
Less than high school graduate	1963 (18.5)	1026 (12.5)	937 (39.3)	<.05	1531 (17.7)	432 (21.8)	<.05
High school graduate	5530 (52.1)	4422 (53.7)	1108 (46.5)	<.05	4373 (50.6)	1157 (58.4)	<.05
College graduate	3126 (29.4)	2788 (33.9)	338(14.2)	<.05	2733 (31.6)	393 (19.8)	<.05
Tobacco dependence, any	542 (5.1)	441 (5.4)	101 (4.2)	≥.05	476 (5.5)	66 (3.3)	<.05
Aggression	3372 (32.1)	2687 (33.0)	685 (29.1)	<.05	2657 (31.1)	715 (36.6)	<.05
Low grade point average	7853 (74.5)	5902 (72.2)	1951 (82.3)	<.05	6141 (71.6)	1712 (86.8)	<.05
Psychological distress	3570 (44.9)	2801 (45.0)	769 (44.6)	≥.05	2949 (45.2)	621 (43.6)	<.05
Chronic medical condition	5027 (47.5)	3983 (48.5)	1044 (44.0)	<.05	4046 (47.0)	981 (49.6)	<.05

^a^ *P* for comparison of Hispanic and non-Hispanic or white and black youth, with *P* < .05 denoting statistical significance.

### Outcomes Based on the Intersections of Race/Ethnicity and Educational Attainment

Table 2 presents the distributions of our binary outcomes based on the intersections of race, ethnicity, and educational attainment. This table shows how the prevalence of our undesired youth outcomes change as a function of educational attainment for each racial/ethnic group.

**Table 2.** Prevalence of Undesired Youth Outcomes by the Intersection of Race/Ethnicity and Educational Level

Outcome	Educational Level															
	Non-Hispanic								Hispanic							
	White, No. (%)			*P* Value^a^	Black, No. (%)			*P* Value^a^	White, No. (%)			*P* Value^a^	Black, No. (%)			*P* Value^a^
	Low	Mid	High		Low	Mid	High		Low	Mid	High		Low	Mid	High	
Tobacco dependence	87 (13.2)	231 (6.9)	65 (2.7)	<.05	17 (4.6)	33 (3.1)	8 (2.2)	<.05	29 (3.3)	52 (5.1)	12 (4.0)	≥.05	2 (3.0)	0 (0.0)	0 (0.0)	≥.05
Aggression	248 (37.9)	1159 (34.8)	629 (26.1)	<.05	150 (41.7)	373 (35.6)	128 (36.3)	≥.05	225 (26.1)	318 (31.7)	78 (26.2)	≥.05	22 (34.4)	31 (32.3)	11 (32.4)	≥.05
Grade point average	548 (84.2)	2524 (75.6)	1285 (53.3)	<.05	333 (91.2)	927 (87.8)	285 (79.8)	<.05	759 (87.7)	820 (81.3)	205 (67.7)	<.05	56 (86.2)	83 (86.5)	28 (84.8)	≥.05
Psychological distress	206 (43.7)	1265 (48.6)	759 (41.0)	≥.05	117 (45.2)	343 (44.7)	111 (41.3)	<.05	243 (40.8)	363 (47.6)	113 (47.1)	≥.05	15 (34.9)	27 (41.5)	8 (40.0)	≥.05
Chronic medical condition	340 (51.7)	1703 (50.8)	1063 (43.9)	<.05	168 (46.0)	519 (49.0)	190 (53.2)	≥.05	337 (38.8)	466 (46.1)	137 (45.7)	≥.05	30 (46.2)	56 (58.3)	18 (51.4)	≥.05

^a^ *P* for trend for comparison of percentage outcome across educational levels within ethnic group, with *P* < .05 denoting statistical significance.

As educational attainment increased, we observed a statistically significant stepwise reduction (*P* < .05 for trend) in the prevalence of tobacco dependence (13.2% vs 6.9% vs 2.7%), aggression (37.9% vs 34.8% vs 26.1%), low GPA (84.2% vs 75.6% vs 53.3%), and CMCs (51.7% vs 50.8% vs 43.9%) for non-Hispanic whites. For whites, psychological distress did not show such a trend (43.7% vs 48.6% vs 41.0%; *P* ≥ .05 for trend). For Hispanic white youth, such a significant stepwise reduction was only seen for low GPA (87.7% vs 81.3% vs 67.7%). For non-Hispanic black youth, tobacco use (4.6% vs 3.1% vs 2.2%), low GPA (91.2% vs 87.8% vs 79.8%), and psychological distress (45.2% vs 44.7% vs 41.3%) showed such a significant stepwise reduction. The same stepwise reduction could not be seen for other outcomes in other racial/ethnic groups (*P* ≥ .05 for trend).

### Multivariable Models

Table 3 presents the summary of 10 logistic regression models in the pooled sample. In all these models, parental educational attainment was the main independent variable. For each outcome, 2 models were run. Although model 1 only entered the main effects of parental educational attainment, race, ethnicity, and covariates, model 2 also included 4 statistical interaction terms between race and ethnicity and parental educational levels (ie, high school graduation and college graduation).

**Table 3.** Summary of 2 Series of Logistic Regressions on the Interactive Effects of Race, Ethnicity, and Parental Educational Attainment on Youth Outcomes

Characteristic	Model 1: Main Effects		Model 2: Model 1 Plus Interactions	
	OR (95% CI)	*P* Value	OR (95% CI)	*P* Value
Tobacco dependence (any)				
Ethnicity (Hispanic)	0.59 (0.46-0.75)	<.001	0.25 (0.16-0.38)	<.001
Race (black)	0.41 (0.31-0.54)	<.001	0.30 (0.18-0.50)	<.001
Male	1.42 (1.19-1.70)	<.001	1.40 (1.17-1.68)	<.001
Age	5.11 (4.08-6.40)	<.001	5.14 (4.11-6.44)	<.001
Parents married	0.53 (0.44-0.64)	<.001	0.54 (0.45-0.65)	<.001
Parental education		<.001		<.001
High school graduate	0.74 (0.59-0.92)	.007	0.50 (0.38-0.66)	<.001
College graduate	0.34 (0.25-0.46)	<.001	0.22 (0.16-0.31)	<.001
Ethnicity (Hispanic) × high school graduate	NA^a^	NA^a^	3.37 (2.00-5.69)	<.001
Ethnicity (Hispanic) × college graduate	NA^a^	NA^a^	5.40 (2.52-11.56)	<.001
Race (black) × high school graduate	NA^a^	NA^a^	1.38 (0.74-2.58)	.31
Race (black) × college graduate	NA^a^	NA^a^	1.93 (0.78-4.78)	.15
Intercept	0.04	<.001	0.06	<.001
Aggression				
Ethnicity (Hispanic)	0.79 (0.71-0.88)	<.001	0.61 (0.49-0.74)	<.001
Race (black)	1.12 (1.00-1.25)	.04	1.18 (0.93-1.50)	.175
Male	1.53 (1.41-1.66)	<.001	1.53 (1.41-1.66)	<.001
Age	1.07 (0.98-1.16)	.12	1.07 (0.98-1.16)	.13
Parents married	0.81 (0.74-0.89)	<.001	0.81 (0.74-0.89)	<.001
Parental education		<.001		<.001
High school graduate	1.00 (0.89-1.12)	.98	0.92 (0.77-1.09)	.32
College graduate	0.74 (0.64-0.84)	<.001	0.62 (0.52-0.75)	<.001
Ethnicity (Hispanic) × high school graduate	NA^a^	NA^a^	1.41 (1.09-1.81)	.008
Ethnicity (Hispanic) × college graduate	NA^a^	NA^a^	1.59 (1.14-2.21)	.006
Race (black) × high school graduate	NA^a^	NA^a^	0.82 (0.62-1.09)	.17
Race (black) × college graduate	NA^a^	NA^a^	1.26 (0.90-1.75)	.18
Intercept	0.47	<.001	0.52	<.001
Low grade point average (other than As)				
Ethnicity (Hispanic)	1.47 (1.30-1.67)	<.001	1.29 (0.97-1.71)	.08
Race (black)	2.20 (1.90-2.55)	<.001	1.54 (1.06-2.24)	.03
Male	2.06 (1.88-2.27)	<.001	2.07 (1.88-2.27)	<.001
Age	1.31 (1.20-1.44)	<.001	1.31 (1.20-1.44)	<.001
Parents married	0.61 (0.55-0.68)	<.001	0.61 (0.55-0.68)	<.001
Parental education		<.001		<.001
High school graduate	0.64 (0.55-0.75)	<.001	0.59 (0.47-0.74)	<.001
College graduate	0.26 (0.23-0.31)	<.001	0.22 (0.18-0.28)	<.001
Ethnicity (Hispanic) × high school graduate	NA^a^	NA^a^	1.05 (0.75-1.46)	.78
Ethnicity (Hispanic) × college graduate	NA^a^	NA^a^	1.41 (0.97-2.06)	.07
Race (black) × high school graduate	NA^a^	NA^a^	1.28 (0.84-1.95)	.23
Race (black) × college graduate	NA^a^	NA^a^	2.00 (1.26-3.17)	.003
Intercept	4.13	<.001	4.64	<.001
Psychological distress				
Ethnicity (Hispanic)	0.98 (0.88-1.11)	.79	0.84 (0.66-1.06)	.14
Race (black)	0.87 (0.77-0.99)	.03	0.95 (0.72-1.26)	.75
Male	0.49 (0.45-0.54)	<.001	0.49 (0.45-0.54)	<.001
Age	1.41 (1.29-1.55)	<.001	1.41 (1.29-1.55)	<.001
Parents married	0.84 (0.76-0.93)	.001	0.84 (0.76-0.93)	.001
Parental education		<.001		<.001
High school graduate	1.27 (1.11-1.44)	<.001	1.22 (1.00-1.48)	.05
College graduate	1.02 (0.88-1.18)	.80	0.93 (0.76-1.14)	.48
Ethnicity (Hispanic) × high school graduate	NA^a^	NA^a^	1.17 (0.89-1.56)	.26
Ethnicity (Hispanic) × college graduate	NA^a^	NA^a^	1.50 (1.05-2.13)	.03
Race (black) × high school graduate	NA^a^	NA^a^	0.85 (0.61-1.17)	.31
Race (black) × college graduate	NA^a^	NA^a^	1.00 (0.68-1.45)	.98
Intercept	0.96	.63	1.02	.86
Chronic medical conditions (any)				
Ethnicity (Hispanic)	0.83 (0.75-0.91)	<.001	0.64 (0.53-0.78)	<.001
Race (black)	0.97 (0.87-1.08)	.55	0.83 (0.66-1.05)	.13
Male	1.43 (1.32-1.55)	<.001	1.43 (1.32-1.54)	<.001
Age	1.05 (0.97-1.13)	.23	1.05 (0.97-1.13)	.25
Parents married	0.73 (0.68-0.80)	<.001	0.74 (0.68-0.80)	<.001
Parental education		<.001		<.001
High school graduate	1.19 (1.07-1.33)	.001	1.04 (0.88-1.22)	.66
College graduate	1.03 (0.91-1.16)	.68	0.83 (0.70-0.98)	.03
Ethnicity (Hispanic) × high school graduate	NA^a^	NA^a^	1.32 (1.05-1.68)	.02
Ethnicity (Hispanic) × college graduate	NA^a^	NA^a^	1.62 (1.20-2.18)	.002
Race (black) × high school graduate	NA^a^	NA^a^	1.06 (0.82-1.39)	.65
Race (black) × college graduate	NA^a^	NA^a^	1.56 (1.14-2.14)	.005
Intercept	0.85	.01	0.98	.85

Abbreviations: NA, not applicable; OR, odds ratio.

^a^ Interaction term was not entered into the model.

According to model 1, parental educational attainment was associated with all youth outcomes, namely tobacco dependence (OR, 0.74 [95% CI, 0.59-0.92]; *P* = .007 for high school graduation; OR, 0.34 [95% CI, 0.25-0.46]; *P* < .001 for college graduation), aggression (OR, 0.74 [95% CI, 0.64-0.84]; *P* < .001 for college graduation), low GPA (OR, 0.64 [95% CI, 0.55-0.75] for high school graduation; OR, 0.26 [95% CI, 0.23-0.31] for college graduation; *P* < .001 for both), psychological distress (OR, 1.27 [95% CI, [1.11-1.44]; *P* < .001 for high school graduation), and CMCs (OR, 1.19 [95% CI, 1.07-1.33]; *P* = .001 for high school graduation). As the direction of these ORs shows, for 3 outcomes, higher parental educational attainment was negatively associated with the undesired youth outcomes (tobacco dependence, aggression, and low GPA), and for 2 outcomes, high parental educational attainment was positively associated with undesired outcome (psychological distress and CMCs).

Model 2 showed statistically significant interactions between race and ethnicity with parental educational attainment on all youth outcomes. Interactions were significant between Hispanic ethnicity and education on tobacco dependence (OR, 3.37 [95% CI, 2.00-5.69] for high school graduation; OR, 5.40 [95% CI, 2.52-11.56] for college graduation; *P* < .001 for both), aggressive behaviors (OR, 1.41 [95% CI, 1.09-1.81]; *P* = .008 for high school graduation; OR, 1.59 [95% CI, 1.14-2.21]; *P* = .006 for college graduation), and psychological distress (OR, 1.50 [95% CI, 1.05-2.13]; *P* = .03). Race showed an interaction with college graduation on low GPA (OR, 2.00 [95% CI, 1.26-3.17]; *P* = .003) and CMCs (OR, 1.56 [95% CI, 1.14-2.14]; *P* = .005). All these findings suggest that the protective effect of high parental educational attainment on youth development might be systemically smaller for Hispanic and black than for non-Hispanic youth. These findings suggested that high parental educational attainment has a less significant association with positive youth outcomes for black and Hispanic families than for non-Hispanic families.

## Discussion

The current study showed significant interactions between race and ethnicity with parental educational attainment on youth outcomes. Overall, the associations between high parental educational attainment and youth outcomes were found to be less significant for ethnic minority families than for non-Hispanic white families. However, this pattern was heterogeneous and inconsistent across ethnic groups and level of education that showed the interaction.

Because MDRs could be observed across outcomes and ethnic minorities (ie, blacks and Hispanics), we hypothesize that the probable causes for the observed MDRs of parental educational attainment are some upstream social forces. This is because proximal or downstream determinants of tobacco dependence, aggression, low GPA, psychological distress, and CMCs vary to a large degree. These outcomes, however, overlap in terms of upstream distal social determinants.^53,54,55^

This study confirmed what was previously found for black adults,^41,42,43^ black youth,^27,28,29^ and Hispanic adults.^44,45,46^ Compared with non-Hispanic white youth, black and Hispanic youth remain at high risk of asthma,^36^ attention-deficit/hyperactivity disorder,^37^ mental health problems,^25^ depression,^30,31^ obesity,^29,35^ dental health problems,^56^ poor health care use,^26^ low GPA,^33^ poor school attainment,^24^ poor school bonding,^34^ impulse control,^27^ and cigarette smoking,^38^ all of which are disproportionate to their high parental education.

These patterns are not limited to youth but carry over into adulthood. These patterns also are not limited to educational attainment^25^ and are seen for income,^42^ employment,^57^ and marital status.^32^ Minorities’ diminished returns are well described for obesity,^29,35^ substance use,^44^ depression,^42^ happiness,^58^ affect,^58^ anxiety,^32^ self-rated health,^59^ CMCs,^37^ oral health,^26^ and mortality.^57^ This study suggests that the same patterns also apply to Hispanic youth.

Because of the existing MDRs, we should observe worse-than-expected outcomes in Hispanic youth from middle-class families. Thus, ethnic health disparities are not limited to the low SES sections of the society because other social mechanisms are at work to diminish the health return of parental education for ethnic minority families.

In this study, Hispanic youth showed less aggression than white youth. This seemingly counterintuitive result could be understood in the context of the Hispanic health paradox^60,61^ and the healthy immigrant effect.^62^ Similarly, black youth had less tobacco dependence than white youth. Some better mental health outcomes of blacks compared with whites have been attributed to resilience, which might be a function of adversity.^63^

As mentioned already, despite the general trend, some inconsistencies were observed. We found differences in MDRs based on race and ethnic minority group, educational level, and the outcome. We need to conduct more studies to understand the circumstances in which a particular educational level does promote an outcome for one but not another ethnic group. Despite these heterogeneities and nuances, black and Hispanic youth tended to be at an overall disadvantage compared with non-Hispanic white youth.

### Future Research

There is a need to study structural and societal factors that result in MDRs of SES indicators for ethnic minority groups. Because diminished returns hold across health outcomes and the health outcomes are universally affected, regardless of the domain, it would appear that some upstream social processes may be interfering with the translation of human capital into health. For example, residential segregation, stressful neighborhoods, unequal quality of schooling, discrimination, and poor access to healthy food options may be mechanisms hindering the development of youth from middle-class ethnic minority families. The high psychological costs of upward social mobility may be another mechanism causing diminished returns of parental educational attainment for ethnic minority youth. At the same time, some mechanisms may be relevant to one but not to other outcomes. For example, predatory tobacco marketing practices may play a role in explaining ethnic differences in returns of SES in the form of tobacco use disparities. However, because these patterns are independent of the outcome, we argue that racism and colorism may be deeper causes. However, testing this hypothesis needs additional research. Again, previous studies^31,64,65^ that have proposed other mechanisms have all been limited to black participants. There is, thus, an urgent need to study the mechanisms of MDRs in Hispanic families. Another unanswered question is the actual magnitude of these MDRs. Some of these interactions were significant but with a small effect. We argue that their upstream nature will have large trickle-down effects, because many outcomes are shaped by more distal social determinants of health. In addition, even though the effect sizes of MDRs may be small, when multiplied throughout the total population larger differences may show up at the population level.^66^

### Policy Implications

These results suggest that specific types of policies and public health programs may be needed to reduce ethnic health disparities. Because disparities are shaped by the differential outcomes of SES, there is a need to go beyond policies focusing exclusively on health to policies addressing the broader social processes placing ethnic minorities at a relative disadvantage. There is a need for innovative national and local policies that reduce disparities due to differential SES and also those that happen at identical SES levels (ie, MDRs).^20,26,27,29,32,41,44,56,67^ There is a need to discover policy and program solutions that equalize a minority population’s abilities to leverage its available educational attainment.^19,20^ Future research should explore the role of discrimination in the labor market,^41^ the potential outcomes of enforcement of existing antidiscrimination laws, and the diminished returns of educational attainment among black and Hispanic families. Communities where ethnic minorities reside may benefit from additional investment aimed at education quality, a strategy that may boost the translation of educational attainment into health outcomes.^68^ Programs that help families secure high-paying jobs are essential. Overall, to undo MDRs of parental educational attainment among Hispanic families, we need to minimize the societal and environmental barriers in the daily lives of ethnic minorities. We should also continuously invest in educational programs aimed at society at large to reduce the unfair treatment of highly educated ethnic minority people.

Policy makers should be aware that inequalities persist among highly educated individuals through diminished returns of human capital among ethnic minority people. This view of health disparities is in line with the view that it is race/ethnicity and class, not race/ethnicity or class, that affect health disparities.^69,70^ This view may alter how we allocate resources to eliminate racial and ethnic health disparities. Instead of a reductionist view that attributes ethnic health disparities solely to SES inequalities, health gaps must be addressed across SES levels. However, this study suggests that simply reallocating resources is not sufficient. We must empower ethnic minorities to efficiently translate their human capital into positive health outcomes, thereby realizing their full health potential.

### Limitations

This study had some methodological limitations. The cross-sectional design prevented us from making any causal inferences. The sample size was imbalanced across racial and ethnic groups. We only included black, Hispanic, and non-Hispanic white youth. Other ethnic minorities such as Asian populations should be included in future studies. We only studied the differential outcome of educational attainment. Other family SES indicators, such as income, employment, and wealth, were not available. These important components of SES are not accounted for in our analyses, and they may confound the associations between education, ethnicity, and youth outcomes. The degree to which disparities remain beyond SES should be determined in the studies that can control for a wide range of SES components. We did not have data on area-level SES factors. This study did not include variation by zip code or geographic location. Despite these limitations, we believe this study still contributes to extending the existing literature on MDRs among US youth. A large sample size, a random sampling, generalizable results to the United States, and the multiple outcomes studied are among the strengths of this study.

## Conclusions

In the United States, black and Hispanic youth are at a relative disadvantage compared with their non-Hispanic white counterparts regarding the magnitude of the association between educational attainment of their parents and youth outcomes.
